# Surgical management of patent ductus arteriosus in pre-term infants - a british paediatric surveillance study

**DOI:** 10.1186/s12887-021-02734-9

**Published:** 2021-06-09

**Authors:** A. Warnock, L. Szatkowski, A. Lakshmanan, L. Lee, W. Kelsall

**Affiliations:** 1 Neonatal Unit, Nottingham University Hospitals NHS Trust, Nottingham, UK; 2 Division of Epidemiology and Public Health, School of Medicine, University of Nottingham, Nottingham, UK; 3 Neonatal Unit, University Hospitals of Coventry and Warwickshire, Coventry, UK; 4 Neonatal Unit, Cambridge University Foundation Trust, Cambridge, CB2 2QQ UK

**Keywords:** Neonatal, Patent Ductus Arteriosus, Ligation, Preterm, Cardiothoracic

## Abstract

**Background:**

This study aimed to provide UK data describing the incidence of patent ductus arteriosus (PDA) surgery in the neonatal population, including: pre-ligation management, and outcomes until discharge. We used British Paediatric Surveillance Unit (BPSU) methodology; collecting data via questionnaires for preterm neonates undergoing PDA ligation (PDAL) between 1st Sept 2012 – 30th Sept 2013. Infants born less than 37 weeks gestation, who underwent PDAL prior to discharge home, with no other structural cardiac abnormality, were included. Information collected included: patient demographics, pre and post-operative clinical characteristics, pre-operative medical management, post-operative complications and outcome.

**Results:**

Over the study, 263 infants underwent PDAL an incidence of 3.07 per 10,000 live births. 88% were born extremely preterm (< 28 weeks) and 60% were male. The commonest reasons for ligation were inability to wean respiratory support (83.7%) and haemodynamically significant PDA (87.8%). Pre-operatively 65.7% received medical therapy. Surgery was performed at a median age of 33 days (range 9-260, IQR 24-48); the corrected age was less than 31 + 6 week in 50.6% babies at PDAL. Most, (90%), of procedures were open ligation; only 9 (3.4%) were catheter occlusions (PDACO). 20.5% of patients had post-operative complications. The 30-day mortality was 3%, with 93.5% surviving to hospital discharge.

**Conclusion:**

This study showed there was little consensus over medical and surgical management of the PDA or timing of surgery.

**Supplementary Information:**

The online version contains supplementary material available at 10.1186/s12887-021-02734-9.

## Background

The patent ductus arteriosus (PDA) remains open at day 7 in 65% of neonates born < 28 weeks’ gestation [1] and in 80% at 24-25 weeks [2]. Haemodynamically significant PDA has been associated with respiratory morbidity including: pulmonary haemorrhage (PH), gastrointestinal and neurological morbidity [3], chronic lung disease (CLD) [4], necrotising enterocolitis (NEC) [5], intraventricular haemorrhage (IVH) [6] and retinopathy of prematurity (ROP) [7]. Failure of PDA closure has been associated with increased mortality [8].

When this study was conceived (2011), there was no consensus regarding neonatal PDA management [9]. There were three main approaches. Firstly, conservative: careful ventilation, fluid administration and possibly diuretics. Secondly, pharmacological closure: cyclooxygenase inhibitors (COXi)/non-steroidal anti-inflammatory drugs (NSAID); Indomethacin or Ibuprofen. There was debate about treatment timing and dosages, with different strategies supported by the medical literature. If medical treatment failed, or was contraindicated surgical ligation was an option. By 2012, use of high dose paracetamol [10] and interventional PDA catheter occlusion (PDACO) [11] was reported, but neither routinely used in UK neonatal practice.

In 2012 there was limited UK data regarding neonatal PDA ligation (PDAL). Retrospective studies [12–14] reported PDAL was well tolerated with good initial survival rates; but higher death rates by 1 year of age. Survivors had high rates of CLD and neuro-disability, thought to be complications of prematurity rather than cardiac surgery. An East of England study [14] estimated national demand for PDAL using the Epicure 2 data set [15], calculating that 133 infants born < 26 weeks gestation would require PDAL annually.

The study aimed to review UK practice of PDAL in preterm neonates to help inform clinicians which neonates might benefit from PDA ligation; highlighting procedure complications, and post-operative morbidity and mortality.

## Methods

### Data collection

A questionnaire (Additional file 1) was sent to all clinicians reporting an infant undergoing PDAL (between 1st Sept 2012 and 30th Sept 2013) through the BPSU “orange card reporting system”, distributed to UK and ROI consultant paediatricians.

Eligible infants were born < 37 weeks’ gestation, underwent PDAL before discharge home, with no other cardiac abnormality. Data included: demographics; pre-operative medical management; clinical status at PDAL; and post-operative complications, including mortality. Missing data, discrepancies and duplicate cases were clarified through contact with reporting clinicians and the CTC (cardiothoracic centre).

### Ethics

Application was made to BPSU in 2011 with permission to proceed in September 2012. Funding was through the Sir Peter Tizard Bursary (held by Dr. Lee). Study ethics approval was granted by NRES Committee - East Midlands - Derby (Reference: ECC 3-02(FT6)/2012. Informed consent from study participants (written or verbal) for use of routinely collected, then anonymized data was not necessary as per BPSU agreement with the national Confidentiality Advisory Group and Health Research Authority.

### Data analysis

Data were transferred into the statistical package Stata v15 (Stata Corp., College Station, Tx) for analysis. Categorical variables were tabulated as frequencies and percentages, missing data was itemised as a separate category. Continuous variables non-normally distributed were summarised using median, range and interquartile range. Chi-squared and Kruskal-Wallis tests were used to assess variations in outcome between patient groups defined by their gestational age and weight, at birth and at PDAL. Logistic regression was used to calculate odds ratios for procedure complications and death according to sex, gestational age at birth and PDAL, weight at birth and PDAL, and the type of closure procedure. *P*-values of < 0.05 were taken to indicate statistical significance. Results were not adjusted for multiple hypothesis testing; instead, we chose to present 95% confidence intervals and *p*-values to allow the reader to judge the evidence for themselves. Using UK birth registration data [16–18] we determined births over the study period to calculate the incidence of PDAL per 10,000 live births.

## Results

**Fig. 1:**
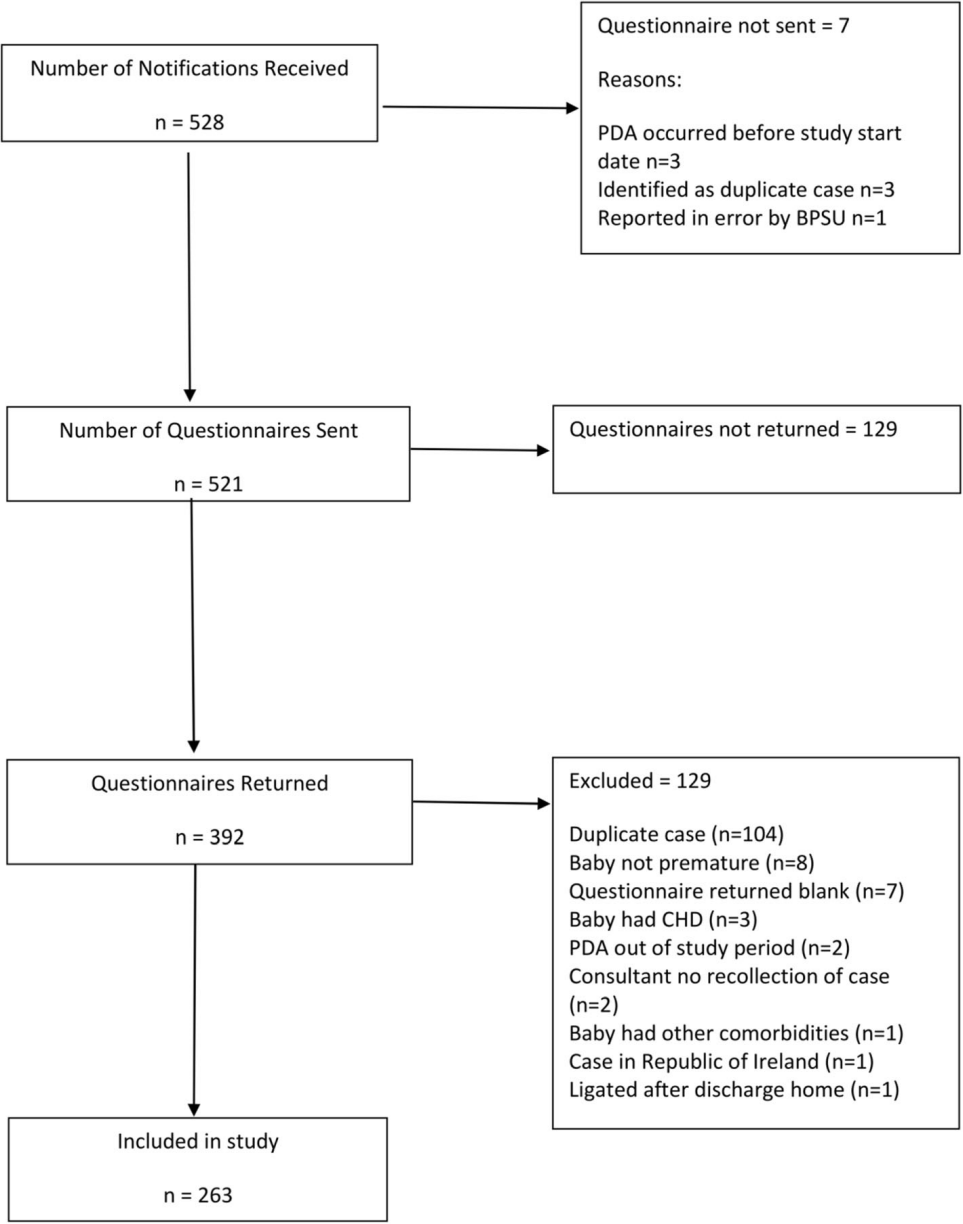
Case identification

Between 1st Sept 2012 to 30th Sept 2013, the BPSU received 528 notifications of neonatal PDAL. Only one response was received from ROI, therefore we concentrated on UK data. After identifying duplicate notifications, 263 babies were included (Fig. 1).

### Incidence

There were 856,794 UK livebirth registrations over the study; the incidence of PDAL was 3.07 per 10,000 births.

### Demographics

Of the 263 babies undergoing PDAL, 231 (88%) were born extremely preterm (< 28 weeks gestation), 60% were male. Two hundred nineteen of the 263 (83%) were extremely low birth weight (≤ 999 g) (Table 1).

**Table 1:** Demographics of neonates undergoing PDA surgery

	n (%)^**a**^
**Gender**	
Male	158 (60.1)
Female	105 (39.9)
**Gestation at Birth**	
Extreme Preterm (< 28 weeks)	231 (87.8)
Very Preterm (28 + 0 - 31 + 6)	25 (9.5)
Moderately Preterm (32 + 0 - 36 + 6)	7 (2.7)
**Birth Weight**	
Extremely low (≤ 999 g)	219 (83.3)
Very low (1000 – 1499 g)	29 (11.0)
Low (1500-2499 g)	6 (2.3)
Normal (≥ 2500 g)	1 (0.4)
Unknown	8 (3.0)
**Ethnicity**	
White	166 (63.1)
Mixed	11 (4.2)
Asian or Asian British	23 (8.8)
Black or British Black	28 (10.7)
Chinese or Other	6 (2.3)
Unknown	29 (11.0)

^a^Numbers do not total 100% due to rounding

### Pre-surgery investigations

Of the 263 infants, 229 (87.1%) had their PDA diagnosed clinically and confirmed by echocardiography (echo) in NICU. Thirty-one (11.8%) PDA’s diagnosed by echo were not suspected clinically based upon answers to the questionnaire (Additional file 1), however this may potentially reflect clinician error in reporting. In 3 cases (1.1%) data was not provided. Eight (3%) infants were reported to have their diagnostic echo in the receiving CTC.

### Pre-surgery clinical features

Common reasons for PDAL were haemodynamic significance (*n* = 231, 87.8%), inability to wean respiratory support (*n* = 220, 84%) and cardiovascular instability (*n* = 81, 31%). Some infants had multiple reasons documented (Table 2). In this study, haemodynamic significance was not based on any pre-defined criteria or measurements and left to the discretion of the reporting clinicians.

**Table 2:** Decision to refer for surgery based on gestational age at birth, with *P* values

Reason for decision to refer for surgery	Number reporting reason for decision to refer for surgery by gestational age at birth (n, %)			Chi^**2**^ ***p***-value for difference between groups
	Extremely preterm (*n* = 231)	Very preterm (*n* = 25)	Moderately preterm (*n* = 7)	
**Inability to wean respiratory support**	199 (86.2)	17 (68.0)	4 (57.1)	0.010
**Haemodynamically Significant**	203 (87.9)	18 (72.0)	6 (85.7)	0.090
**Cardiovascular instability**	73 (31.6)	6 (24.0)	2 (28.6)	0.730
**Poor growth**	48 (20.8)	11 (44.0)	5 (71.4)	< 0.001
**Contra-indication of medical therapy**	44 (19.1)	3 (12.0)	0 (0)	0.312
**Other**^**a**^	31 (13.4)	3 (12.0)	3 (42.9)	0.083

^**a**^ Other reasons (*n* = 37) included: Unable to complete medical treatment (*n* = 12), NEC (*n* = 8), PH (*n* = 4), feeding difficulty (*n* = 2), duct thought not to be related to prematurity (*n* = 1), evolving aortic stenosis (*n* = 1), RSV negative bronchiolitis (*n* = 1), pulmonary hypoplasia (*n* = 1), congestive cardiac failure (*n* = 1), thrombus in aorta (*n* = 1), chromosome disorder (mosaic trisomy 14) (*n* = 1). 4 questionnaires specified “other” but no further explanation was documented

Extremely preterm and lower birth weight infants were more likely to be referred for inability to wean respiratory support, *p* = 0.010 and *p* = 0.042 respectively. Moderately preterm infants were referred because of poor growth (*p* < 0.001) (Table 2).

### Pre-surgery medical management

One hundred and seventy three (65.7%) of the cohort received medical treatment before referral. The majority (*n* = 143, 82.6%) received targeted therapy, 3 infants had prophylactic treatment. In 27 cases (15.6%), there was insufficient information. Ibuprofen was most frequently used (*n* = 148, 85.6%) with 11 (6.4%) receiving Indomethacin. Seven infants received both drugs. In 7 cases there was no information. Almost half of treated babies (*n* = 83, 48%) received one course of Ibuprofen; 42% (*n* = 73) received 2 courses, and one received 3 courses. Treatment was completed in 122 (70.5%) cases. The commonest reasons for discontinuing treatment were deranged renal function (35.5%, *n* = 11) and suspected sepsis (22.6%, *n* = 7). Seventy four (28.1%) babies were not treated medically, of these 56 (75.7%) had several reasons documented, with renal impairment being the most frequent (24.3%) (Table 3).

**Table 3:** Reasons for not using NSAID pre-surgery

	n (%)
**Renal Impairment**	18 (24.3)
**Thrombocytopenia**	12 (16.2)
**Sepsis**	13 (17.6)
**NEC**	12 (16.2)
**IVH**	4 (5.4)
**> 21 days old when decision to treat made**	13 (17.6)
**Abdominal Concerns (bilious aspirate/concern re. NEC/Feed intolerance)**	9 (12.2)
**Pulmonary Haemorrhage**	2 (2.7)
**Spontaneous Gut Perforation**	2 (2.7)
**Other**	6 (8.1)

Other reasons (*n* = 6) included: not indicated, limb ischemia, exomphalos major, pericardial effusion, minimal support needed, expectation PDA would close without pharmacological treatment

### Morbidity pre- PDAL

#### Neurology

Overall 106 (40.3%) babies had IVH and 13 (4.9%) of the whole cohort had parenchymal involvement. Eighteen (6.8%) had documented ROP pre-surgery. Unsurprisingly, extremely preterm infants were more likely to have IVH prior to surgery (*p* = 0.015).

#### Respiratory

Two hundred and one (76.4%) infants remained mechanically ventilated pre-PDAL, with 124 (61.7%) having an oxygen requirement > 40%. Thirty-eight (14.4%) were receiving CPAP, with 7 (18.4%) having an oxygen requirement > 40%, consistent with significant compromise. PH was reported in 30 (11.4%) babies.

#### Cardiovascular

Inotropes were being administered to 26 (9.9%) infants, and fluid restriction (based on local practice) was part of the medical management in 102 (38.8%) infants at PDAL. There were no significant differences by gestation or birth weight.

#### Nutrition

NEC was confirmed radiologically in 70 (26.7%) infants pre-PDAL. Almost half the babies (*n* = 125, 47.5%) were on full feeds, 10 (3.8%) on full parenteral nutrition (PN), with 77 (29.2%) receiving some feeds and PN at referral. In 51 cases, no data was provided. The more premature and lower birth weight infants were more likely to be receiving PN at the time of surgery.

### Referral pathway

Of the 263 infants referred for PDAL, 199 (75.7%) required ambulance transfer to a CTC. Of these, 129 (64.8%) had been discussed with one CTC and 25 (12.5%) with two or three centres to arrange PDAL. No information was available for 45 (22.5%) babies. Of the whole cohort, 59 (22.4%) babies were in a NICU co-located with a CTC. In 5 cases, data was unavailable.

The interval between referral and PDAL was median 6 (0-42, IQR 3-9) days. Distance travelled was a median of 15 (0 – 123, IQR 2-30) miles, longest distances were in Scotland.

### Surgical characteristics

Surgery was performed at median age of 33 (9-260, IQR 24-48) days and median weight 1010 (500-4000, IQR 845.5-1410) grams. At PDAL, 133 (50.6%) babies had a corrected age between 28 and 31 + 6 weeks, and 113 (43%) were < 1 kg. Of the 199 patients transferred to a CTC, 42.2% were classified as a day case procedure. This could mean admission and discharge on same day, or may include one overnight stay.

### Surgical procedure

**Fig. 2:**
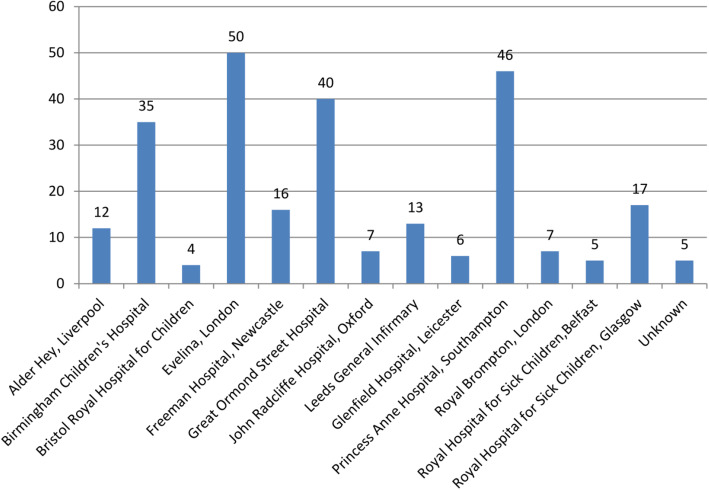
Bar graph illustrating number of PDA surgeries undertaken by cardiothoracic centre

Open ligation was performed in 237 (90%) neonates, including one following an unsuccessful PDACO. PDACO was successfully performed in 9 (3.4%) infants with median weight 2060 (558 – 3388, IQR 1900 - 2620) grams. Method of PDA closure was not documented in 17 (6.5%) cases. All procedures were conducted in a CTC, with 60.8% undertaken in theatre and 29.7% performed on a co-located NICU. In 9.5% of cases this information was unavailable. Procedures were conducted in 13 CTCs, the median number was 13 (4-50, IQR 6-35) (Fig. 2).

### Length of intubation post-procedure

Perioperative ventilation data was reported for 192 of the 283 (73%) babies in the cohort and showed median time from PDAL to extubation of 5 days (0-46, IQR 2-12). Babies who had always been intubated pre-PDAL were extubated after a median of 7 days (1-46, IQR 1-41). Those electively intubated for the procedure were extubated after median of 2 days (0-7, IQR 1-5). Extremely preterm infants took longer to extubate (*p* < 0.001). There was no significant difference in time to extubation between neonates undergoing open ligation compared to PDACO.

### Post-operative complications

Post-operative complications occurred in 54 (20.5%) cases, most frequent were: pneumothorax (*n* = 25, 9.5%), vocal cord palsy (*n* = 6, 2.3%), wound infection (*n* = 6, 2.3%) and chylothorax (*n* = 2, 0.8%) (See additional file 2 for full list). Two infants (0.8%) died on the second post-operative day, in neither was death directly attributed to PDAL. There were no significant differences in complication rates by sub-group analysis (Additional file 3). We did not collect post-procedure information from the CTC regarding post-PDAL syndrome including: haemodynamic instability, inotrope use, blood product administration, ventilator instability or oxygen requirements.

### Morbidity post- surgery

Of the 263 infants, 132 (50.2%) developed CLD, defined as need for supplementary oxygen at 36 weeks corrected, 85 (64.4%) received diuretics and 29 (22%) post-natal steroids. Only 18 (13.6%) required no additional medical treatments. Extremely low birth weight babies were more likely to require medical treatment (steroids *p* = 0.041 and diuretics *p* = 0.035). 32 (12.2%) infants required laser treatment for ROP following ligation. 12 (4.6%) infants developed further episodes of NEC requiring surgery. No further episodes of PH were documented. There were no significant differences in post-operative co-morbidities by gestational age.

### Mortality

There were 17 (6.5%) deaths. For 16 patients the interval between PDAL and death was available, median 33 (2-239, IQR 10-89) days. Eight infants died within 30 days of surgery giving an overall 30-day mortality of 3%. There were no significant differences in mortality rates by sub-group analysis (Additional file 4).

## Discussion

This was the first UK wide study to investigate the management of preterm infants undergoing PDAL. Our inclusion criteria were designed to capture neonates failing medical management or where it was contraindicated. In 2012-2013, there was no consensus about optimal management of PDA, timing of medical treatment or when PDA ligation should be considered. These controversies are unchanged in 2021. We believe our study provides important information about PDAL, which is still relevant today. The study highlights the characteristics of babies referred, and provides outcome information about morbidity and survival.

In our study, PDA ligation occurred in 3.07 per 100,000 live births. We note that the prevalence of PDAL in premature babies will be higher, but did not have access to preterm denominator data in order to calculate ligation rates accurately in this population. We also did not have contemporaneous access to the NICOR (National Institute for Cardiovascular Outcomes Research) database, which provides information about all UK PDA closures, not just in the neonatal population [19]. Comparisons with NICOR data are difficult, as our study ran over 2 NICOR reporting years, 2012/13 and 2013/14. Their dataset classifies neonates as < 30 days and an infant as 31 days to 1 year of age [20]. Over half of our cohort might be classified as infants, with a median age of 33 days at PDAL, making it almost impossible to cross-reference numbers. Their data identified only a small number of PDACO, consistent with our observations. Subsequent NICOR data up to 2016 (Additional file 5) suggests fewer neonates are undergoing PDA closure, but it will be difficult to apply this to the preterm population because of their classification criteria.

Our study again highlights that a higher proportion of extremely preterm males with extremely low birth weight, and ethnically classified as white, require PDAL; correlating with other published series [21–24]. The majority of infants in this study were reliant on mechanical ventilation prior to surgery, with 62% needing > 40% oxygen, consistent with significant respiratory compromise. It is a limitation of our study that we did not more accurately define haemodynamic significance in terms of PDA diameter, left atrium: aorta ratios or blood flow patterns. Many of the infants required ambulance transfer with the additional resources and challenges this poses. Some CTCs performed more surgical procedures than others, which may simply reflect the geographical population they serve, but could signify different referral practices for surgical intervention. Such variations are likely to continue until there is a greater consensus regarding management of the neonatal PDA.

The overall mortality rate in our study was 6.5%, with a 30-day mortality rate of 3%. It is reassuring that PDA surgery appears a relatively safe procedure in this challenging patient population. Complication rates were recorded as 20.5%, most such as pneumothoraces and wound infections were easily treated. Vocal cord palsies and chylothoraces should be considered more chronic complications.

The most common pre-surgical co-morbidities were IVH and NEC, in keeping with published literature [25, 26]. It has been suggested that the incidence of CLD increases following PDA surgery [4, 12–14, 25, 26]. In our cohort, 50% of babies had CLD, but in reality, this will be an underestimate as 28% had either not reached 36 weeks corrected age or had died by study close. As this was a nationwide questionnaire based study, we could not identify case-control groups to compare whether rates of CLD and other complications were statistically higher following surgical closure, which is a limitation of our observational study.

It is important to consider whether this study is relevant to neonatologists in 2021. In 2014, Heuchan et al. [27], reported that the optimal management of the PDA in premature infants remained controversial, as did Sankar et al. [28] in 2019. Over the 7 years since this study ended there have been many advances in neonatal care: including the wider use of paracetamol [29]; different evidence-based approaches to the management of respiratory distress syndrome [30]; and new techniques for less invasive surfactant administration (LISA) [31] all of which may have contributed to fewer neonates and infants requiring PDA closure, as suggested by the NICOR data. Despite these advances, PDA management remains controversial. There is still no consensus on whether to, or when to treat pharmacologically. Many clinicians now accept a PDA, treating fewer medically and limiting PDAL to babies with worsening respiratory failure. There are ongoing worldwide trials assessing the benefits of early versus no pharmacological treatment: the OSCAR Study [32], Beneductus Trial [33] and EPAR Trial [34]. The recent international PDA-TOLERATE pilot exploratory trial [35] with 202 infants, showed no difference in PDA ligation rates between the control and treatment groups. Small numbers of neonates will continue to require PDA closure with increasing evidence that transcatheter closure is being safely used [36].

## Conclusion

Our study provides important information on approaches to PDA management across the UK in 2012/2013. Extremely preterm and extremely low birth weight male infants were more likely to undergo PDAL due to failed pharmacological treatment and / or ongoing invasive ventilation requirement. This study suggests that PDAL has a high survival rate (93.5%), with the majority of patients (80%) suffering no complications. It also suggests a potential link between PDA surgery and CLD needing on-going treatment.

This study confirmed there was variation in medical management used prior to referral for PDAL. This variation persists in 2021 with ongoing debate regarding appropriate management strategies. If our BPSU study was conducted again today, we might find more neonates had been treated medically with Paracetamol, and of those referred for PDA closure, a greater proportion might undergo catheter occlusion.

This study should stimulate the neonatal community to consider standardising the medical and surgical management of neonatal PDA. Getting it right first time (GIRFT) neonatal network reviews should help to identify best practice and identify outliers.

## Supplementary Information

**Additional file 1.** BPSU Questionnaire

**Additional file 2.** Post-operative complications. Number of patients presenting with a post-operative complication

**Additional file 3.** Post-operative complication sub-group analysis. Post-operative complications sorted by gestational age and weight

**Additional file 4.** Mortality sub-group analysis. Mortality rates sorted by gestational age and weight

**Additional file 5.** NICOR data. NICOR data by year

## Data Availability

The datasets used and/or analysed during the current study are available from the corresponding author on reasonable request. The datasets supporting the conclusions of this article are included within the article (and its additional files).

## Abbreviations

PDA: Patent Ductus Arteriosus
BPSU: British Paediatric Surveillance Unit
PDAL: PDA Ligation
PDACO: PDA Catheter Occlusion
PH: Pulmonary haemorrhage
CLD: Chronic Lung Disease
NEC: Necrotising Enterocolitis
IVH: Intraventricular Haemorrhage
ROP: Retinopathy of Prematurity
COXi: Cyclooxygenase inhibitor
NSAID: Non-steroidal anti-inflammatory drug
CTC: Cardiothoracic centre
ROI: Republic of Ireland
NICU: Neonatal Intensive Care Unit
CPAP: Continuous Positive Airway Pressure
echo: echocardiograph
PN: Parenteral nutrition
NICOR: National Institute for Cardiovascular Outcomes Research
