# Supplementary material for: Surgical management of patent ductus arteriosus in pre-term infants - a british paediatric surveillance study
Source: BMC Pediatr. 2021 Jun 9;21:270. doi: 10.1186/s12887-021-02734-9 (PMC8187455; doi:10.1186/s12887-021-02734-9)
Supplement: Supplementary file 1 — Additional file 1. BPSU Questionnaire [file 12887_2021_2734_MOESM1_ESM.pdf]

Case notification form - Strictly Confidential

Surgically Ligated Patent Ductus Arteriosus in Premature Babies

The first page of the case notification form will be stored separately from the rest of the questionnaire and personal identifying information for the case will be used only for linkage of records.

SECTION 1: REPORTER DETAILS

1.1 Date of completion of questionnaire: : _ _ / _ _ / _ _ _ _

1.2 Consultant responsible for case at your hospital ....

1.3 Name of your hospital ....

1.4 Telephone number:

1.5 Email address:

1.6 Has the patient been referred to/from another centre?

Yes ☐ No ☐ Unknown ☐

If yes:

a) Please name centre ....

b) Please name consultant ....

1.7 Name of hospital at which PDA closure undertaken ....

1.8 Location of PDA closure

NICU ☐ Cardiothoracic Theatre ☐ Other ☐ Please specify ....

SECTION 2: CHILD'S DETAILS

2.1 Date of birth: _ _ / _ _ / _ _ _ _

2.2 NHS / CHI number: ....

2.3 Hospital Number at your hospital ....

2.4 Sex: Male ☐ Female ☐

2.5 Gestation at birth: ……..weeks and ………..days

2.6 Birth Weight. ………. grams

2.8 Ethnicity*: ☐☐ * please see table in Appendix A

2.10 Please specify other ethnicity here …………………………………………………

SECTION 3: DECISION TO REFER FOR PDA LIGATION:

3.1 Hospital giving care at time of decision to ligate PDA ………………………………

3.2 Hospital Number at hospital in 3.1: ……………………………….

3.3 Was diagnosis made by: Clinical examination ☐ Echocardiography ☐ *Please tick all that apply*

3.4 If ECHO performed, where was this? *Please tick all that apply*

Referring hospital ☐

At the centre where PDA ligation was performed ☐

Other institutions ☐ *please specify*…………………….

3.5 Was decision to ligate based on: *please tick all that apply*

Inability to wean respiratory support Yes ☐ No ☐

Need for postnatal steroids for chronic lung disease Yes ☐ No ☐

Cardiovascular instability Yes ☐ No ☐

Poor growth Yes ☐ No ☐

Contraindication of medical therapy Yes ☐ No ☐

Haemodynamic significance on echocardiography Yes ☐ No ☐

Other ☐ *please specify* …………………………………….

SECTION 4: PATENT DUCTUS ARTERIOSUS SPECIFIC TREATMENT BEFORE PDA LIGATION:

4.1 Was a NSAID eg ibuprofen/indomethacin used pre-ligation? Yes ☐ No ☐

If No, please go to section 4.5.

If Yes, please continue.

If yes, please indicate which was used:

Indomethacin Yes ☐ No ☐ Unknown ☐

Ibuprofen Yes ☐ No ☐ Unknown ☐

4.2 Was the treatment

Prophylactic Yes ☐ No ☐ If yes go to 4.3

Targeted Yes ☐ No ☐ If yes go to 4.4

4.3 For Prophylactic treatment

What dose was given? ……….mg/kg/day for ………….days

Was the course completed? Yes ☐ No ☐

4.4 For targeted treatment

What dose was given? …………mg/kg/day for ……..days

Then ……………..mg/kg/day (if appropriate) for …..days

How many courses were given? …..courses

Were the courses completed? Yes ☐ No ☐ Unknown ☐

If not completed, please state why…………………..

4.5 If indometacin or ibuprofen were not used, was the reason documented?

Yes ☐ No ☐

4.6 If documented was the reason:

Renal impairment	Yes ☐	No ☐	Unknown ☐
Thrombocytopaenia	Yes ☐	No ☐	Unknown ☐
Sepsis	Yes ☐	No ☐	Unknown ☐
Other	Yes ☐		

Please specify other ………………………………………………………………………………………

SECTION 5: MORBIDITY BEFORE PDA LIGATION

Before ligation, did the baby have: (please tick all that apply)

Radiologically confirmed necrotising enterocolitis	Yes ☐	No ☐
Necrotising enterocolitis requiring surgery	Yes ☐	No ☐
Intraventricular haemorrhage	Yes ☐	No ☐
Pulmonary haemorrhage	Yes ☐	No ☐
Intraventricular haemorrhage with infarction	Yes ☐	No ☐
Retinopathy of prematurity	Yes ☐	No ☐

SECTION 6: CLINICAL PICTURE ON DAY BEFORE LIGATION:

6.1 Respiratory support *(tick which option is appropriate)*

Intubated and ventilated ☐ FiO2 ……………%

If intubated and ventilated, was this only for transfer for PDA ligation?

Yes ☐ No ☐

If yes, please also tick the mode of respiratory support prior to intubation for transfer

CPAP ☐ FiO2 ……………%

Nasal cannula oxygen ☐ FiO2……………..l/min ☐ or % ☐

None ☐

6.2 Cardiovascular support

Inotropes Yes ☐ No ☐ Not known ☐

Fluid restricted Yes ☐ No ☐ Not known ☐

6.3 Nutrition

Full enteral feeds Yes☐ No ☐ Not known ☐

Less than full enteral feeds Yes☐ No ☐ Not known ☐

Parental Nutrition Yes☐ No ☐ Not known ☐ Not Applicable☐

SECTION 7: LIGATION PROCEDURE:

7.1 Please tick which procedure was used to close the PDA

Open Ligation ☐ Catheter occlusion ☐

Please answer the whole questionnaire for all PDA closures – whether ligation or catheter occlusion

7.2 Date of referral for PDA ligation _ _ / _ _ / _ _ _ _

7.3 Number of institutions contacted to perform ligation………

7.4 Date of PDA ligation _ _ / _ _ / _ _ _ _

7.5 At the time of ligation what was the baby's:

Age ………days

Weight ……….grams

7.6 Was the ligation performed as a day case? Yes ☐ No ☐

7.7 Date of extubation **post surgery** _ _ / _ _ / _ _ _ _ or still ventilated ☐

SECTION 8: POST OPERATIVE SURGICAL COMPLICATIONS

Were there any complications related to surgery? Yes ☐ No ☐

If yes please tick all that apply

Chylothorax ☐

Pneumothorax ☐

Wound infection ☐

Death ☐

Other ☐ please specify ……………………………………………

SECTION 9: CO-MORBIDITY POST PDA LIGATION

9.1 Does/did the baby have chronic lung disease (defined as oxygen requirement at 36 weeks corrected gestational age) Yes ☐ No ☐ Not applicable ☐

(*Not applicable if not yet 36 weeks corrected gestational age)*

9.2 Post ligation, did the baby have:

Postnatal steroids for chronic lung disease?	Yes ☐	No ☐
Diuretics for chronic lung disease?	Yes ☐	No ☐
Retinopathy of prematurity requiring laser treatment	Yes ☐	No ☐
Radiologically confirmed necrotising enterocolitis	Yes ☐	No ☐
Pulmonary haemorrhage	Yes ☐	No ☐
Necrotising enterocolitis requiring surgery	Yes ☐	No ☐
Intraventricular haemorrhage filling ventricle	Yes ☐	No ☐
Intraventricular haemorrhage with parenchymal infarct	Yes ☐	No ☐

SECTION 10: MORTALITY

Has baby died? Yes ☐ No ☐

If yes, what was the date of death? _ _ / _ _ / _ _ _ _

What was the cause of death? …………………………………………………………..
