# Supplementary material for: Surgical management of patent ductus arteriosus in pre-term infants - a british paediatric surveillance study
Source: BMC Pediatr. 2021 Jun 9;21:270. doi: 10.1186/s12887-021-02734-9 (PMC8187455; doi:10.1186/s12887-021-02734-9)
Supplement: Supplementary file 2 — Additional file 2. Post-operative complications. Number of patients presenting with a post-operative complication [file 12887_2021_2734_MOESM2_ESM.pdf]

	**n (%)***
**Pneumothorax**	25 (9.5)
**Wound Infection**	6 (2.3)
**Vocal Cord Palsy**	6 (2.3)
**Hypotension**	5 (1.9)
**Renal Failure**	3 (1.1)
**Chylothorax**	2 (0.8)
**Death**	2 (0.8)
**Limb Hypoperfusion**	2 (0.8)
**Lung collapse/consolidation**	2 (0.8)
**Other****	15 (5.7)

Supplementary Material 2 - Numbers of patients with a documented complication post-operatively

*percentages being out of the whole cohort of patients to show the global chance of having this complication when a patient undergoes PDA surgery.

** "other" reasons (all n=1) were: stridor, tricuspid regurgitation, persistent left ventricular dilatation, sagittal sinus and left axillary thrombosis, thoracic duct occlusion, systemic inflammatory response, phrenic nerve palsy, suspected sepsis, NEC, pulmonary hypertension, pulmonary tears x2, aortic tear and post-ligation syndrome. One patient’s trans-catheter occlusion "failed", therefore they were transferred to another centre for a ligation procedure. One patient had a "PDA not fully clipped i.e. still some flow".
