# Supplementary material for: Surgical management of patent ductus arteriosus in pre-term infants - a british paediatric surveillance study
Source: BMC Pediatr. 2021 Jun 9;21:270. doi: 10.1186/s12887-021-02734-9 (PMC8187455; doi:10.1186/s12887-021-02734-9)
Supplement: Supplementary file 3 — Additional file 3. Post-operative complication sub-group analysis. Post-operative complications sorted by gestational age and weight [file 12887_2021_2734_MOESM3_ESM.pdf]

	n	Reporting any complication (n, %)	Unadjusted odds ratio for complication (95% CI)	p-value
**Gender**				
Male	158	35 (22.2)	1.0	0.426
Female	105	19 (18.1)	0.78 (0.42-1.45)	
**Gestation at Birth**				
Extremely preterm	231	47 (20.4)	1.0	0.869
Very preterm	25	5 (20.0)	0.98 (0.35-2.74)	
Moderately preterm	7	2 (28.6)	1.57 (0.29-8.33)	
**Birth Weight**				
Extremely low (≤ 999g)	219	46 (21.0)	1.0	0.678
Very low (1000 – 1499g)	29	5 (17.2)	0.78 (0.28-2.17)	
Low (1500-2499g)	6	2 (33.3)	1.88 (0.33-10.6)	
Normal (≥ 2500g)	1	1 (100)	Omitted	
Unknown	8	0 (0)	Omitted	
**Gestation at procedure**				
Extremely preterm	35	10 (28.6)	1.0	0.447
Very preterm	133	30 (22.6)	0.73 (0.31-1.68)	
Moderately preterm	49	7 (14.3)	0.42 (0.14-1.23)	
Term +	37	6 (16.2)	0.48 (0.15-1.51)	
Unknown	9	1 (11.1)	0.31 (0.03-2.83)	
**Weight at procedure**				
≤ 999g	113	28 (24.8)	1.0	0.480
1000 – 1499g	77	11 (14.3)	0.51 (0.23-1.09)	
1500-2499g	41	8 (19.5)	0.74 (0.30-1.78)	
≥ 2500g	17	3 (17.7)	0.65 (0.17-2.43)	
Unknown	15	4 (26.7)	1.10 (0.33-3.74)	
**Ethnicity**				
White	166	36 (21.7)	1.0	0.210
Mixed	11	4 (36.4)	2.06 (0.57-7.44)	
Asian or Asian British	23	2 (8.7)	0.34 (0.08-1.54)	
Black or British Black	28	5 (17.9)	0.79 (0.28-2.21)	
Chinese or Other	6	3 (50.0)	3.61 (0.70-18.7)	
Unknown	29	4 (13.8)	0.58 (0.19-0.40)	
**Type of procedure**				
Open ligation	236	46 (19.5)	1.0	0.318
Catheter occlusion	9	3 (33.3)	2.07 (0.50-8.57)	

Supplementary Material 3 - Odds ratio for having any complication by gestational age, gestation and weight at surgery, ethnicity and type of procedure.
