# Supplementary material for: Surgical management of patent ductus arteriosus in pre-term infants - a british paediatric surveillance study
Source: BMC Pediatr. 2021 Jun 9;21:270. doi: 10.1186/s12887-021-02734-9 (PMC8187455; doi:10.1186/s12887-021-02734-9)
Supplement: Supplementary file 4 — Additional file 4. Mortality sub-group analysis. Mortality rates sorted by gestational age and weight [file 12887_2021_2734_MOESM4_ESM.pdf]

	n	Deaths (n, %)	Unadjusted odds ratio for death (95% CI)	p-value
**Gender**				
Male	158	10 (6.3)	1.0	0.913
Female	105	7 (6.7)	1.06 (0.39-2.87)	
**Gestation at Birth**				
Extremely preterm	231	15 (6.5)	1.0	0.774
Very preterm	25	2 (8.0)	1.25 (0.27-5.82)	
Moderately preterm	7	0 (0)	Omitted	
**Birth Weight**				
Extremely low (≤ 999g)	219	14 (6.4)	1.0	0.433
Very low (1000 – 1499g)	29	3 (10.3)	1.69 (0.45-6.27)	
Low (1500-2499g)	6	0 (0)	Omitted	
Normal (≥ 2500g)	1	0 (0)	Omitted	
Unknown	8	0 (0)	Omitted	
**Gestation at procedure**				
Extremely preterm	35	2 (5.7)	1.0	0.851
Very preterm	133	10 (7.5)	1.34 (0.28-6.42)	
Moderately preterm	49	0 (0)	Omitted	
Term +	37	4 (10.8)	2.0 (0.34-11.7)	
Unknown	9	1 (11.1)	2.1 (0.17-25.7)	
**Weight at procedure**				
≤ 999g	113	8 (7.1)	1.0	0.736
1000 – 1499g	77	4 (5.2)	0.72 (0.21-2.48)	
1500-2499g	41	3 (7.3)	1.04 (0.26-4.11)	
≥ 2500g	17	0 (0)	Omitted	
Unknown	15	2 (13.3)	2.02 (0.39-10.5)	
**Ethnicity**				
White	166	9 (5.4)	1.0	0.665
Mixed	11	1 (9.1)	1.74 (0.20-15.17)	
Asian or Asian British	23	3 (13.0)	2.62 (0.65-10.47)	
Black or British Black	28	1 (3.6)	0.65 (0.08-5.31)	
Chinese or Other	6	1 (16.7)	3.49 (0.37-33.09)	
Unknown	29	2 (6.9)	1.29 (0.26-6.31)	
**Type of procedure**				
Open ligation	236	16 (6.8)	1.0	N/A
Catheter occlusion	9	0 (0)	Omitted	

Supplementary Material 4 - Odds ratio for mortality by gestational age, gestation and weight at surgery, ethnicity and type of procedure.
