# Supplementary material for: Surgical management of patent ductus arteriosus in pre-term infants - a british paediatric surveillance study
Source: BMC Pediatr. 2021 Jun 9;21:270. doi: 10.1186/s12887-021-02734-9 (PMC8187455; doi:10.1186/s12887-021-02734-9)
Supplement: Supplementary file 5 — Additional file 5. NICOR data. NICOR data by year [file 12887_2021_2734_MOESM5_ESM.pdf]

	Catheter closure			Surgical closure			Grand Total
Year	Neonate[1]	Infant[2]	Total	Neonate[1]	Infant[2]	Total	
2015-16	1	107	108	74	183	257	365
2014-15	1	129	130	94	180	274	404
2013-14	1	120	121	130	206	336	457
2012-13	1	159	160	137	230	367	527
2011-12	1	134	135	147	240	387	522
2010-11	1	111	112	177	273	450	562

[1] Neonates 1-30 days of age, [2]Infant 31-365 days of age

Supplementary Material 5 - PDA closure rates NICOR data 2010 - 2016
